# Characterization of Apoptosis, Autophagy and Oxidative Stress in Pancreatic Islets Cells and Intestinal Epithelial Cells Isolated from Equine Metabolic Syndrome (EMS) Horses

**DOI:** 10.3390/ijms19103068

**Published:** 2018-10-08

**Authors:** Katarzyna Kornicka, Agnieszka Śmieszek, Jolanta Szłapka-Kosarzewska, Jennifer M. Irwin Houston, Michael Roecken, Krzysztof Marycz

**Affiliations:** 1 Department of Experimental Biology, The Faculty of Biology and Animal Science, Wroclaw University of Environmental and Life Sciences, 50-375 Wrocław, Poland; kornicka.katarzyna@gmail.com (K.K.); smieszek.agnieszka@gmail.com (A.Ś.); jolanta.szlapka@gmail.com (J.S.-K.); d.weiss@horsedoc.ch (J.M.I.H.); 2 PferdePraxis Dr. Med. Vet. Daniel Weiss, Postmatte 14, CH-8807 Freienbach, Switzerland; 3 Faculty of Veterinary Medicine, Equine Clinic-Equine Surgery, Justus-Liebig-University, 35392 Gießen, Germany; MRoecken@t-online.de

**Keywords:** equine metabolic syndrome, horses, intestinal cells, pancreatic islets, autophagy

## Abstract

Endocrine disorders are becoming an increasing problem in both human and veterinary medicine. In recent years, more and more horses worldwide have been suffering from equine metabolic syndrome (EMS). This metabolic disorder is characterized by pathological obesity, hyperinsulinaemia, hyperglycaemia and insulin resistance. Although metabolic disorders, including diabetes, have been extensively studied, there are still no data on the molecular effects of EMS in horses. Thus, the aim of this study was to evaluate apoptosis, oxidative stress, autophagy and microRNA (miR) expression in multipotent intestinal epithelial stem cells (IECs) and pancreatic islets (PIs) isolated post mortem form healthy and EMS diagnosed horses. Our group was the first to describe how EMS affects IEC and PI aging and senescence. First, we evaluated isolation and culture protocol for these cells and subsequently established their metabolic status in vitro. Both IECs and PIs isolated from EMS horses were characterized by increased apoptosis and senescence. Moreover, they accumulated elevated levels of reactive oxygen species (ROS). Here we have observed that autophagy/mitophagy may be a protective mechanism which allows those cells to maintain their physiological function, clear protein aggregates and remove damaged organelles. Furthermore, it may play a crucial role in reducing endoplasmic reticulum (ER) stress. This protective mechanism may help to overcome the harmful effects of ROS and provide building blocks for protein and ATP synthesis.

## 1. Introduction

Equine metabolic syndrome (EMS) has become one of the most frequent endocrine disorders in large animal veterinary medicine, especially in modern and developed countries [1]. Excessive weight, insulin resistance and laminitis are the cardinal signs of EMS phenotype, and all, when not properly treated, lead to devastation of the body and sometimes necessitate euthanasia. EMS is a complex disease, the causes of which can be found, *inter alia*, in the excessive supply of nutrients and reduced physical activity, which lead to insulin and glucose dysregulation, elevated systemic inflammation and pathological adiposity [2,3,4]. Moreover, adipose tissue inflammation leads to the deterioration of adipose stromal stem cells (ASCs), impairing their physiological functions and causing their senescence [5,6,7]. Additionally, abundant infiltration of adipose tissue by peptide hormones, such as leptin, or several cytokines including adiponectin, visfatin, plasminogen activator inhibitor-1 (PAI-1) and apelin, makes adipose tissue not only an energy storage tissue but an active endocrine organ which affects energy homeostasis, glucose metabolism and immune response.

The role of the pancreas per se seems to be fundamental in EMS development, and plays a regulatory role in further disease progression or regression. The molecular and functional homeostasis between beta and alpha cell secretory activities maintains glucose/insulin balance, which ensures normoglycaemia instead of hyperglycaemia. Moreover, the loss of beta cell mass, which may be caused by progressive apoptosis, increases the progressive deterioration of their action. Beta cells have been reported as one of the crucial cells in type 2 diabetes T2D development, and recent data indicate that beta cells are the first line cells that are damaged much earlier before the clinical signs of diabetes [8,9]. This indicates the importance of these cells in the early T2D diagnosis. It seems that beta cell failure, which in turn leads to pancreas dysfunction, occurs on multiple levels, starting with compensation, dysfunction and finally leading to irreversible failure. It was proposed that even single beta cell defects can lead to whole organ damage, as it entails a whole cascade of events which, as a chain reaction, impairs the function of neighbouring beta cells.

Increased insulin production in turn prompts beta cell stress, as the cells are not able to cope with elevated insulin demand leading to the onset of diabetes [10]. Reduction in both beta cell mass and function contributes to the pathogenesis of beta cell failure. Furthermore, it has been proven that increased apoptosis contributes to beta cell loss in T2D. However, the mechanisms that underlie the progressive development of beta cell failure during the course of T2D are not currently fully understood [11,12]. Additional models of cell death have been described in regards to pancreatic failure. One involves autophagy, i.e., a self-digesting mechanism responsible for organelle removal characterized by massive vacuolization. Normally, autophagy is a beneficial, protective mechanism that is markedly up-regulated during starvation or growth factor deficiency, as it helps maintain the functions of cell structures and provide energy for cell survival. On the other hand, conspicuous autophagy can act as a trigger of apoptosis referred to as type 2 programmed cell death [13]. It has also been shown that altered autophagy may be implicated in diseases such as hepatic encephalopathy, neurodegenerative disorders, cancer and potentially T2D development. Recent studies have demonstrated that autophagy is fundamental for maintaining beta cell architecture and function, but its alternations are also involved in beta cell apoptosis. An increased number of “balloon-like” beta cells, with many ultrastructural abnormalities such as mitochondrial swelling and cisternal distension of ER and Golgi apparatus, was observed in Atg-7-deficient mice [14,15,16]. Interestingly, it is still unclear whether autophagy plays a protective or harmful role in diabetes. Our own research has indicated that autophagy serves as a protective mechanism in adipose-derived stem cells isolated from EMS individuals, because it helps maintain their stemness and differentiation potential [17,18,19,20]. Similarly, both autophagy and mitophagy help maintain homeostasis in insulin-sensitive tissues in EMS horses [21].

The main function of beta cells is to maintain glucose homeostasis through insulin synthesis and secretion. Insulin biosynthesis accounts for 50% of total protein synthesis in stimulated beta cells, and the endoplasmic reticulum (ER) plays a key role in this process. Secreted proteins are correctly folded and assembled by chaperones in the ER lumen. However, during insulin resistance, when insulin production is significantly elevated both the ER and chaperones become overloaded and do not retain their function, which leads to ER stress. Stressful conditions can be overcome through several mechanisms which together are known as the unfolded protein response (UPR), which involves enlarging the ER size and increased clearance of misfolded proteins. Recent results indicate that ER stress is one of the molecular mechanism leading to beta cell damage during T2D [22]. ER stress triggered by nitric oxide (NO) in MIN6 cells leads to the induction of apoptosis through the activation of C/EBP homologous protein (CHOP) [23]. On the other hand, in vivo mutations in eukaryotic translation initiation factor 2 alpha kinase 3 (PERK) prevent the development of proper UPR. This excess insulin needs to be degraded by beta cells to maintain relatively constant number of beta granules. Therefore, autophagy is essential to preserve optimal insulin storage during dysfunctional insulin secretion. However, whether and to what extent ER stress plays a role in PI and IEC dysfunction in EMS is largely unknown.

Beta cells, in addition to ER stress, are also prone to oxidative stress, mainly because of low levels of antioxidants such as superoxide dismutase (SOD), catalase and glutathione peroxidase. Lenzen et al. [24] reported that the levels of cytoplasmic Cu/ZnSOD and mitochondrial MnSOD were in the range of 30–40% of the levels in the liver. Oxidative stress may lead not only to the accumulation of misfolded proteins, but also cause damage to mitochondria. Moreover, production of reactive oxygen species (ROS) induced by metabolic stress represents a common injury pathway in the cascade of events that ultimately results in beta cell failure. Accumulating evidence indicates that obesity and hyperglycaemia are associated with increased ROS production. If they are not rapidly eliminated, ROS can damage mitochondria by DNA fragmentation, protein crosslinking, peroxidation of membrane phospholipids and by activating a series of stress pathways eventually leading to cell death [25,26].

The intestinal mucosa absorbs essential nutrients from the lumen to the body. Moreover, it produces mucous and cytokines with protective and signaling properties. Rapid tissue renewal is driven by a pool of multipotent intestinal epithelial stem cells (IECs) that are critical in maintaining the absorptive function of the gut as well as the protective epithelial barrier [27]. Several studies have demonstrated the propagation of IECs from rodents and humans. Although IECs isolated from horse intestines are highly desirable for biomedical and clinical applications, such as toxicity testing, drug development and food adjectives testing, no data exist about equine IEC propagation in in vitro culture. Moreover, it is worth assessing whether and how EMS affects IEC populations, which may consequently lead to disease progression and improper nutrient absorption. 

Therefore, the present study investigated the effect of EMS on PIs and IECs, and whether autophagy, ER and oxidative stress could contribute to its dysfunction. We examined several features of PIs and IECs in EMS, including morphological, functional and gene expression characteristics.

## 2. Results

### 2.1. Expression of Surface Antigens in IECs

**Figure 1:**
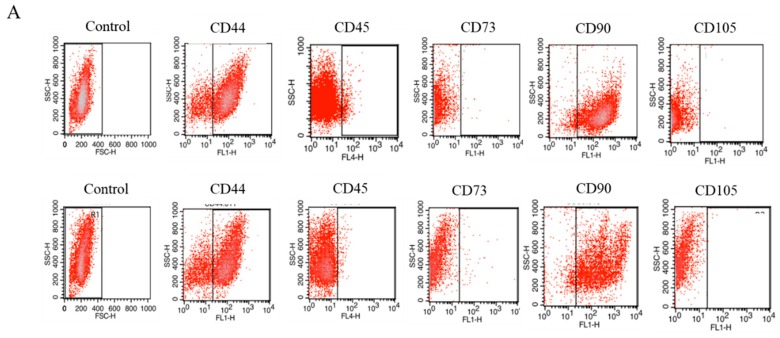

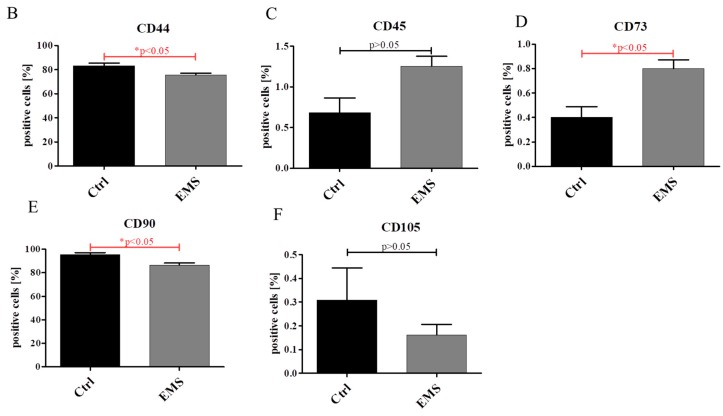
Flow cytometry analysis of IECs phenotype (**A**). Isolated cells are characterized by the presence of CD44 (**B**) and CD90 (**E**) markers. The low expression was noted for CD45 (**C**), CD73b (**D**) and 105 (**F**). Comparative analysis revealed a statistically significant difference in the expression of CD44, CD73 and CD90. The increased percentage of CD44 and CD90 cells was noted in an IEC population isolated from control/healthy horses. The IECs_EMS_ were characterized by the highest number of CD73 positive cells. Results are expressed as mean ± S.D. * *p* < 0.05.

Using flow cytometry, the expression of the following surface antigens in IECs (Figure 1A presents dot plot graphs) was investigated: CD44 (Figure 1B), CD45 (Figure 1C), CD73 (Figure 1D), CD90 (Figure 1E) and CD105 (Figure 1F). IECs in both groups were characterized by the lack of CD45, CD73 and CD105 expression. Only CD44 and CD90 antigens were highly expressed by those cells, although in IECs_EMS_ this expression was downregulated in comparison to control group. 

### 2.2. Morphology and Proliferation of IECs and PIs in Culture

**Figure 2:**
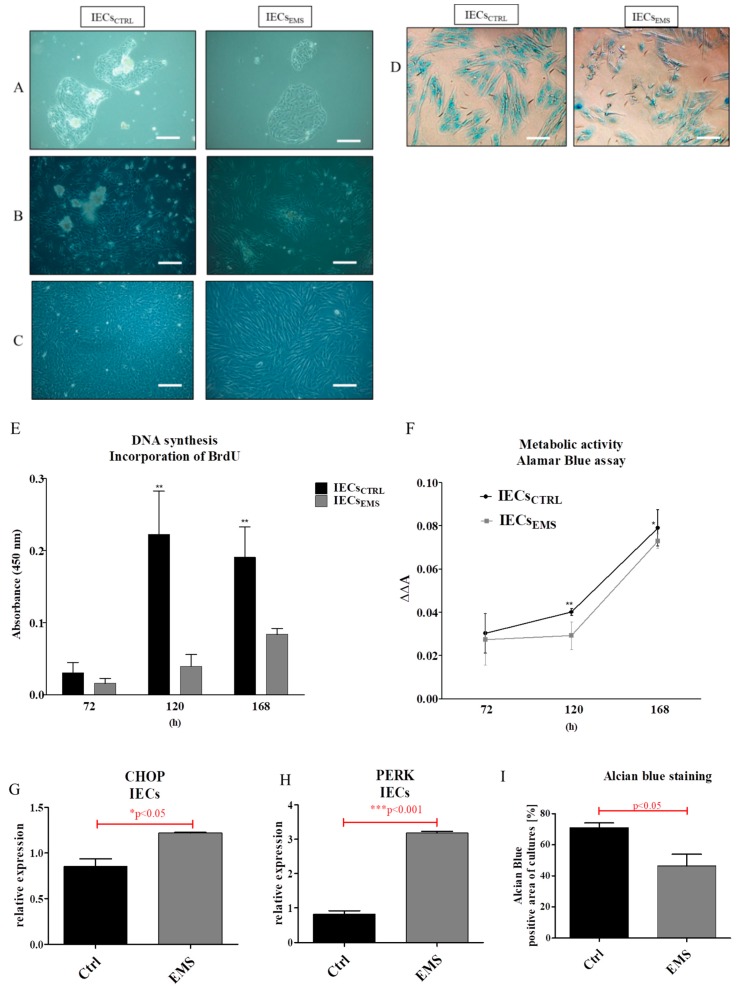
Morphology and proliferation of IECs in subsequent cultures (P1). The cultures were monitored after 72 (**A**), 120 (**B**) and 168 (**C**) h after passaging and stained with Alcian Blue to confirm mucin secretion (**D**). DNA synthesis was established using BrdU assay (**E**) and cell number during the cultivation was assessed with Alamar Blue (**F**). Moreover, using RT-PCT we evaluated the expression of ER stress related genes including CHOP (**G**) and PERK (**H**). Additionally, Alcian Blue staining quantification was performed using ImageJ, which revealed decreased mucin formation in IECs_EMS_ (I). Scale bars: light microscope 100 µm. Results expressed as mean ± S.D. * *p* < 0.05; ** *p* < 0.01; *** *p* < 0.001.

Cell morphology was imaged with light microscope during the culture after 72 (Figure 2A), 120 (Figure 2B) and 168 (Figure 2C) h, which indicated the lower proliferation rate of IECs_EMS_. Alcian Blue staining for mucin was also performed (Figure 2D). The proliferation of cells was established after 72, 120 and 168 h of culture with BrdU assay (Figure 2E). IECs_CTRL_ were characterized by increased proliferation in comparison to EMS cells after 120 and 168 h of propagation. Similar results were obtained from the Alamar Blue assay, which indicates metabolic activity of cells (Figure 2F). CHOP (Figure 2G) and PERK (Figure 2H) expression was elevated in IECs_EMS_. Quantification of Alcian Blue staining revealed decreased mucin formation in IECs_EMS_ (Figure 2I).

**Figure 3:**
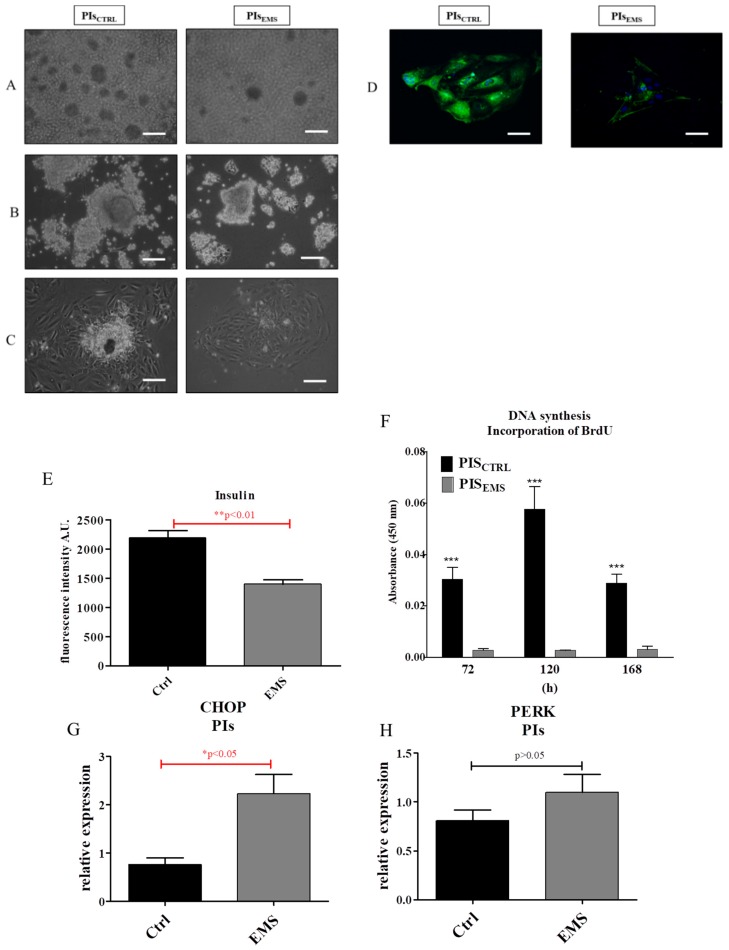
Morphology and proliferation of PIs in culture. The cultures were monitored after 72 (**A**), 120 (**B**) and 168 (**C**,**D**) h in culture. Immunofluorescence staining for insulin (**E**) was quantified using ImageJ by the meaning of fluorescence intensity. The obtained data indicate decreased insulin secretion by PIs_EMS_ (**F**). Proliferation of cells was established using BrdU assay which indicates DNA synthesis rate. In the control group we observed increased BrdU incorporation in comparison to EMS, where DNA synthesis remained at a similar level during propagation. Additionally, using qRT-PCR we established the expression of two genes involved in ER stress. The amount of both CHOP (**G**) and PERK (**H**) mRNA was increased in PIs_EMS_, but only CHOP showed a statistically significant difference (*p* < 0.05). Scale bars: light microscope 400 µm; confocal microscope 40 µm. Results expressed as mean ± S.D. * *p* < 0.05, ** *p* < 0.01; *** *p* < 0.001

Images from light microscope obtained after 72 and 120 h of cultures showed decreased islet size from EMS pancreas (Figure 3A,B) in comparison to control. Moreover, after 168 h, most of the dose cells attached to the culture dish surface, forming small aggregates (Figure 3C). Immunofluorescence staining revealed both decreased islet size and insulin expression in PIs_EMS_ (Figure 3D). The intensity of insulin fluorescence was quantified with the ImageJ software, showing decreased insulin production in EMS (Figure 3E). Proliferation of cells was established with BrdU incorporation assay, which indicates the frequency of DNA replication, after 96, 120 and 168 h of propagation (Figure 3F). At each time, cells from control group proliferated at the greatest rate, while EMS was characterized by constant growth kinetics. Moreover, using qRT-PCR the expression of ER stress-related proteins including CHOP (Figure 3G) and PERK (Figure 3H) was established. Interestingly, both transcripts were elevated in EMS, but only CHOP showed statistically significant difference.

### 2.3. Assessment of Senescence and Apoptosis in IECs and PIs Cultures

**Figure 4:**
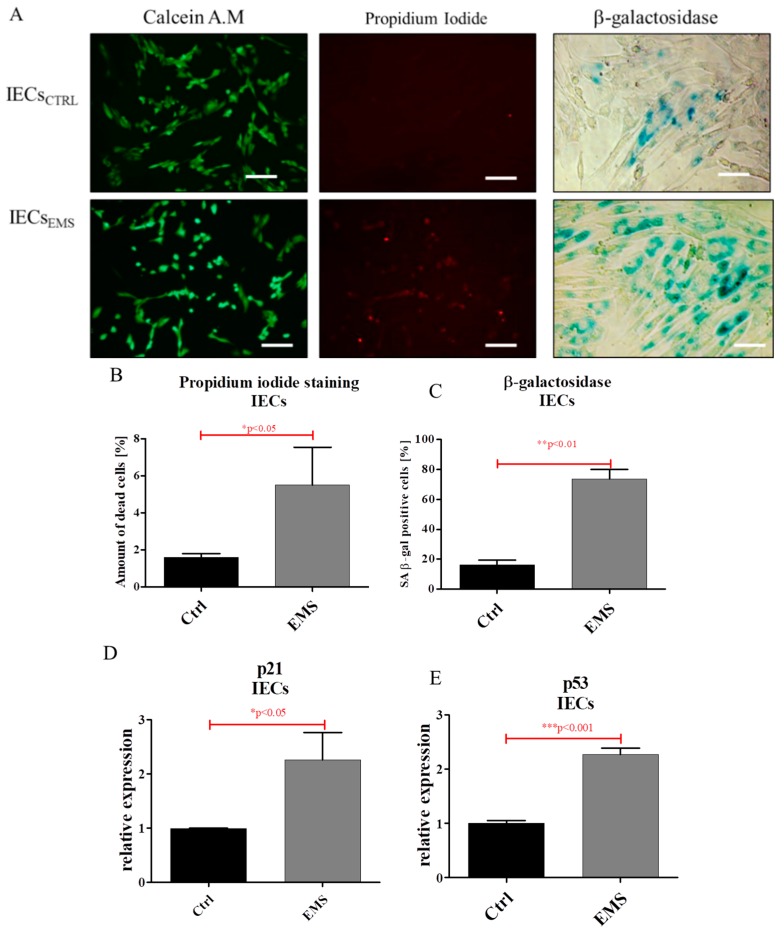
Evaluation of apoptosis and senescence in IECs_CTRL_ and IECs_EMS_. Fluorescence staining of live/dead cells and accumulation of β-galactosidase (**A**). Quantification of images acquired with calcein/propdium iodide (**B**) and senescence stainings (**C**). Using RT-PCR, expression of following transcripts was investigated for p21 (**D**) and p53 (**E**). Scale bars: 400 µm. Results expressed as mean ± S.D. * *p* < 0.05, ** *p* < 0.01, *** *p* < 0.001.

Using fluorescence microscopy, we visualized viable, dead and senescent cells in cultures (Figure 4A). Quantification of calcein AM/propidium iodide revealed an increased number of dead cells in IECs_EMS_ (Figure 4B). Similar results were obtained in β-galactosidase staining (Figure 4C). Moreover, we observed increased expression of both p21 (Figure 4D) and p53 (Figure 4E) in IECs_EMS_.

**Figure 5:**
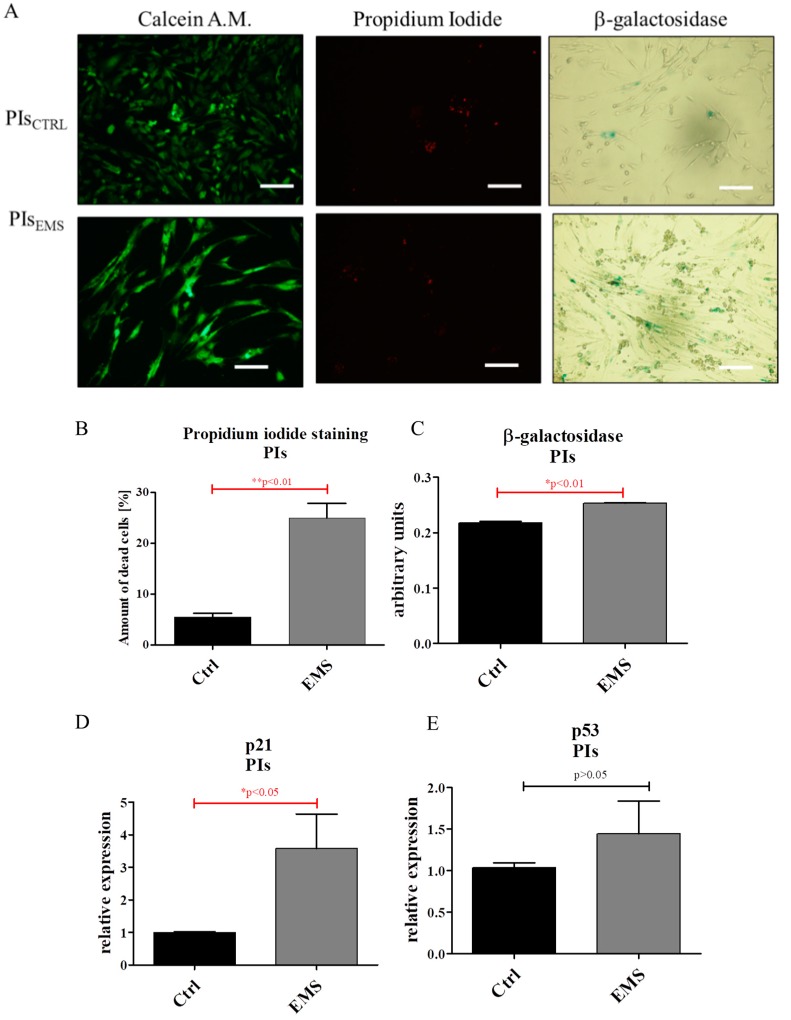
Assessment of senescence and apoptosis in PIs. Using epifluorescence imaging live cells were visualized with calcein while nuclei of apoptotic cells were visualized with propidium iodide (**A**). Based on the representative pictures the percentage of dead cells was calculated for both investigated populations. The obtained data clearly indicate an increased number of dead cells in PIs_EMS_ (**B**). We also established the accumulation of senescence associated β-galactosidase (**A**). Using ImageJ, data from images was quantified (**C**), revealing elevated dye aggregation in EMS group. Similarly, the expression of both p21 and p53 genes was increased in that group (**D**,**E**), however only p21 expression was upregulated significantly. Scale bars: 400 µm. Results expressed as mean ± S.D. * *p* < 0.05, ** *p* < 0.01,

Using the fluorescent dyes calcein and propidium iodide, the amount of viable and apoptotic cells in cultures as well as accumulation of senescence associated β-galactosidase was visualized (Figure 5A Based on the representative pictures, the percentage of dead cells was subsequently calculated, showing an increased number of apoptotic cells in PIs_EMS_ (Figure 5B). Moreover, qRT-PCR analysis revealed increased levels of the apoptotic related genes p21 (Figure 5D) and p53, however without statistical significance (Figure 5E), in PIs_EMS_.

### 2.4. Autophagy in IECs and PIs

**Figure 6:**
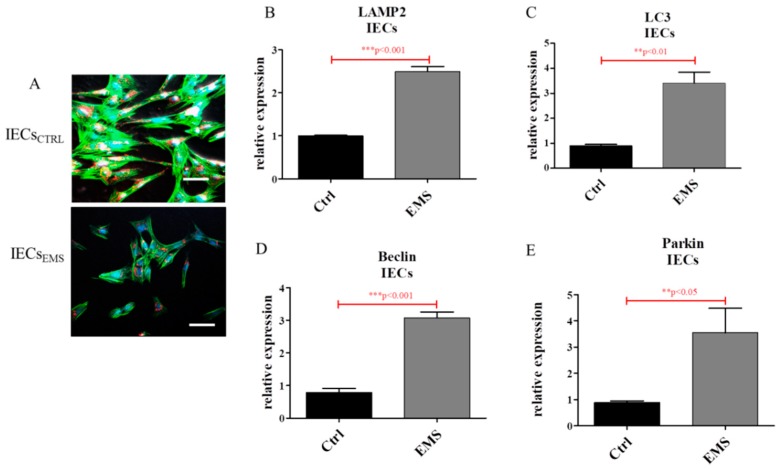
Autophagy in IECs_CTRL_ and IECs_EMS_. Mitochondria condition and autophagy in IECs. Using confocal microscopy, co-localization of LAMP2 and mitochondria was visualized in investigated cultures (**A**). Representative photographs indicate increased lysosome and mitolysosome formation in EMS groups. Moreover, RT-PCR analysis revealed increased expression of auto/mitophagy related genes including LAMP2 (**B**), LC3 (**C**), Beclin (**D**) and Parkin (**E**). Scale bars: confocal microscope: 20 µm. Results expressed as mean ± S.D. ** *p* < 0.01, *** *p* < 0.001.

Staining IECs for mitochondria revealed decreased mitochondria number in IECs_EMS_ (Figure 6A). Furthermore, the expression of LAMP2 (Figure 6B), LC3 (Figure 6C), Beclin (Figure 6D) and Parkin (Figure 6E) was up-regulated in IECs_EMS_.

**Figure 7:**
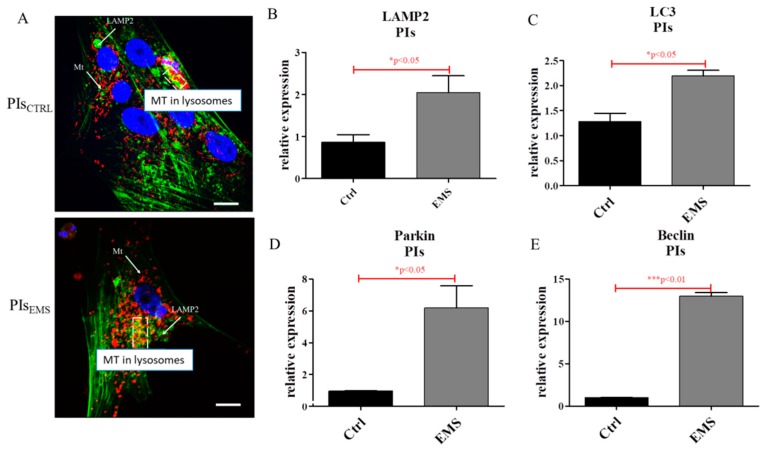
Autophagy in PIs. Using confocal microscopy, co-localization of LAMP2 and mitochondria was visualized in investigated cultures (**A**). Representative photographs indicate increased lysosome and mitolysosome formation in EMS groups. Moreover, using qRT-PCR, the expression of autophagy-related genes including LAMP2 (**B**), LC3 (**C**), Parkin (**D**) and Beclin (**E**) was evaluated. The obtained data confirmed increased autophagy in PIs_EMS_. Scale bars: confocal microscope: 5 µm. Results expressed as mean ± S.D. * *p* < 0.05, *** *p* < 0.001.

Using confocal microscopy, the morphology of PIs including mitochondria and lysosomes was visualized. Representative pictures revealed increased formation of lysosomes and mitolysosomes in PIs_EMS_ (Figure 7A). Autophagy was further investigated by measuring mRNA levels for LAMP2 (Figure 7B), LC3 (Figure 7C), Parkin (Figure 7D) and Beclin (Figure 7E). Each of these genes was up-regulated in PIs_EMS_, which indicates increased autophagy in those cells. 

### 2.5. Oxidative Stress Factors in IECs and PIs

**Figure 8:**
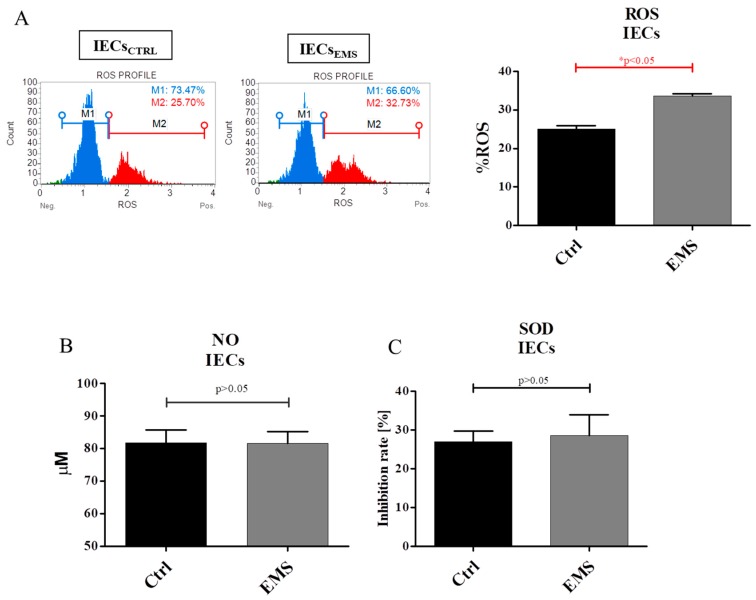
Oxidative stress factors in IECs cultures. The amount of ROS was elevated in IECs_EMS_ in comparison to control group (**A**), whereas NO amount analysis showed no significant differences between groups (**B**). Similarly, the activity of the antioxidative enzyme SOD (C) was comparable in IECs_CTRL_ and IECs_EMS_. Results expressed as mean ± S.D. * *p* < 0.05.

The oxidative stress in IECs_EMS_ was significantly elevated; we observed that those cells accumulate a greater amount of ROS in comparison to control group (Figure 8A). Interestingly, no differences were observed between IECs_CTRL_ and IECs_EMS_ in the case of NO amount (Figure 8B) and SOD activity (Figure 8C).

**Figure 9:**
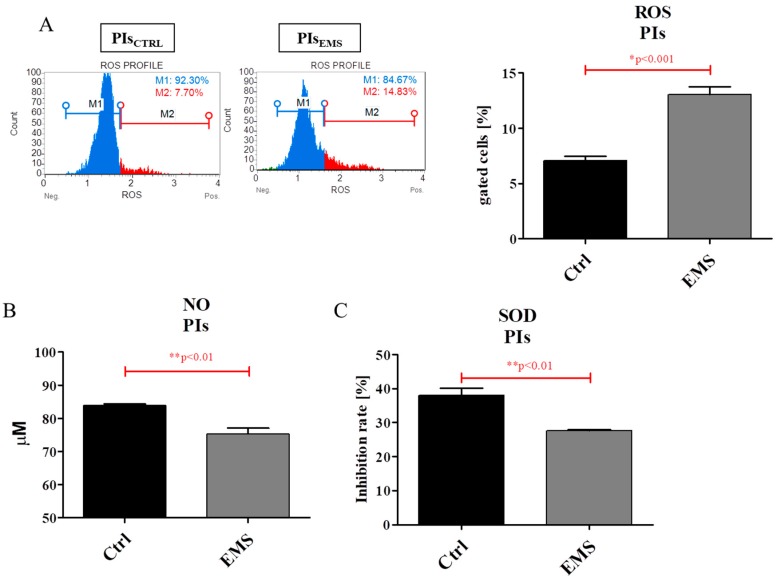
Oxidative stress factors in PIs. Using a MUSE Cell Analyzer, the amount of ROS was established (**A**). Moreover, using colorimetric assays, both NO (**B**) and SOD (**C**) levels were evaluated. The obtained results indicate increased accumulation of oxidative stress in PIs_EMS_, with simultaneously decreased antioxidative capacities. Results expressed as mean ± S.D. * *p* < 0.05.

The obtained data clearly indicate increased accumulation of ROS in PIs_EMS_ in comparison to control group (Figure 9A). Similarly, the amount of NO was also observed to be increased in those cells (Figure 9B). However, conversely, the activity of the antioxidative enzyme SOD was observed to be diminished in PIs_EMS_ (Figure 9C).

### 2.6. miR Expression in IECs and PIs

**Figure 10:**
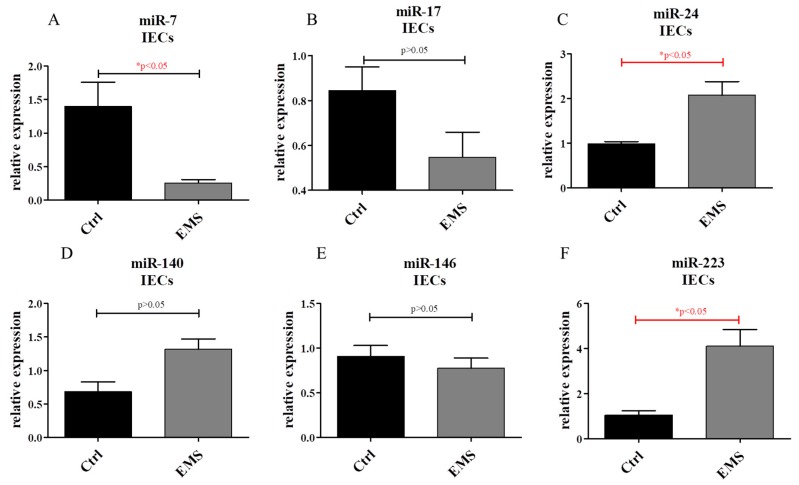
miR expression in IECs. Using PCR, the expression of the following miRNAs was investigated: miR-7 (**A**); miR-17 (**B**); miR-24 (**C**); miR-140 (**D**); miR-146 (**E**); and miR-223 (**F**). Results expressed as mean ± S.D. * *p* < 0.05.

PCR reaction allowed miR expression to be established in IEC cultures. We evaluated the expression of miR-7 (Figure 10A), miR-17 (Figure 10B), miR-24 (Figure 10C), miR (Figure 10D), miR-146 (Figure 10E) and miR-223 (Figure 10F). Interestingly, only miR-24 and miR-223 levels showed a statistically significant difference, with both being upregulated in IECs_EMS_. Conversely, miR-7 was significantly down-regulated in that group (Figure 10A).

**Figure 11:**
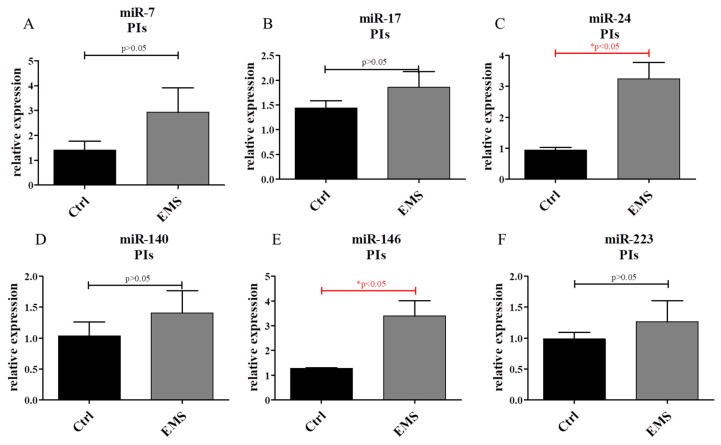
miR expression in PIs. Using PCR, the expression of the following miRNAs was investigated: miR-7 (**A**); miR-17 (**B**); miR-24 (**C**); miR-140 (**D**); miR-146 (**E**); and miR-223 (**F**). Results expressed as mean ± S.D. * *p* < 0.05.

Using PCR, the expression of miRs in PIs was established. No differences in expression of miR-7 (Figure 11A) and miR-17 (Figure 11B) was noted between groups. However, miR-24 (Figure 11C) was found to be up-regulated in PIs_EMS_. Only miR-7 and miR-24 had a statistically significant difference. The expression of miR-140 (Figure 11D), miR-146 (Figure 11E) and miR-223 (Figure 11F) was found to be simultaneously increased in PIs_EMS_, although only miR-146 was up-regulated significantly in comparison to control group. 

## 3. Discussion

In recent years, metabolic disorders have become a rapidly growing problem in both human and veterinary medicine. As recently reported by The World Horse Welfare organization, approximately 50% of riding horses in the UK suffer from obesity [28]. Thus, the aim of this study was to investigate whether and how EMS affects the condition, senescence and mitochondria of the PIs and IECs of horses suffering from EMS in comparison to healthy individuals. 

Hyperglycaemia and metabolic control have always been associated with defects in beta cells in both T1D and T2D [29,30]. Those abnormalities were observed in many models, including humans and rodents, however no data exist for horses [31,32]. Apoptosis may be triggered by many stimuli, however it is executed by two major programs downstream of the death signal: the caspase pathway and mitochondrial dysfunction [33]. Despite some evidence of glucose toxicity leading to cell death [34], the molecular mechanisms underlying this phenomenon are still unclear. It has been shown in PIs that increased apoptosis leads to beta cell mass loss, and consequently, to the deterioration of pancreatic function [9]. In our study, we observed an increased number of both dead and senescent cells in PIs_EMS_. Moreover, the levels of pro-apoptotic genes, including p21 and p53, were also up-regulated, which indicated increased apoptosis of PIs isolated from EMS horses and a negative effect of hyperglycaemia on their survival rate. Interestingly, EMS seemed to exert similar effects on IEC cells, as we observed higher apoptosis of IECs isolated from EMS individuals. Therefore, we postulate that improper balance of endocrinological signalling molecules that occurs in EMS may contribute to both PI and IEC deterioration. These results are consistent with our previous research, in which we observed increased apoptosis in liver and adipose tissue in EMS horses [21].

The relevance of ER stress in the development of diabetes has been extensively studied, as it can induce beta cell death [23] as well as insulin resistance [22]. However, it has not yet been elucidated whether ER stress actually occurs in T2D or whether it contributes to disease development. Increased insulin biosynthesis under high glucose conditions can overwhelm the ER folding capacity, causing an imbalance in homeostasis and leading to ER stress. Thus, in the present study, we evaluated the expression of PERK and CHOP, as they are responsible for developing the unfolded protein response (UPR) during ER stress [35]. Karaskow et al. [36] showed that UPR activation might significantly contribute to palmitate-induced, pancreatic beta-cell death. In our study, we observed up-regulation of both PERK and CHOP in PIs_EMS_ and IECs_EMS_.

Autophagy is a dynamic, self-degradation process, which plays a housekeeping role in the clearance of misfolded or aggregated proteins and damaged organelles, including mitochondria and ER [37]. It has been reported that protein aggregates formed in beta cells during oxidative stress were associated with hyperglycaemia and were regulated by autophagy [8]. Moreover, decreased function and mass of beta cells of Atg7Δ beta cell mice might have been caused by mitochondrial dysfunction and ER stress, because these organelles are rejuvenated by autophagy and are critical for beta cell function and survival [12,38]. In the present study, we observed similar phenomena: PIs_EMS_ were characterized by increased expression of both autophagy- and mitophagy-related genes. Thus, we speculate that autophagy functions as a protective mechanism in these cells, helping to eliminate mitochondria deteriorated by ROS and preserve cell functions.

Many studies have indicated the negative role of ROS in T2D development and progression [25,39]. ROS overload can exceed cell antioxidant capacity, leading to oxidative stress [40]. It has been proven that prolonged exposure to hyperglycaemia causes non-enzymatic glycation of proteins, leading to ROS production [41]. PIs are particularly susceptible to ROS damage, because the expression of antioxidant enzymes in these cells is extremely low [26]. It was demonstrated by Sakuraba et al. [42] that type 2 diabetic patients showed a reduction of beta-cell mass and increased oxidative stress-related tissue damage. It was also confirmed in a rodent model that the cyclin-dependent kinase inhibitor p21 was induced by oxidative stress, and increased levels were found in pancreatic islet cells upon diabetes development [43]. Another study showed that elevated free fatty acids or glucose levels increased apoptosis in rat pancreatic islets, and these cytotoxic effects could be mediated by oxidative stress. This could contribute to the beta-cell failure that occurred in most T2D patients a few years after the onset of clinical diabetes [44]. Our data seem to be consistent with the observations made in humans and rodents, as we have recorded increased ROS and decreased SOD activities in PIs isolated from EMS subjects. Interestingly, some studies have indicated the possibility of ameliorating PI oxidative stress with antioxidant treatments. For example, Lee et al. observed that feeding db/db mice with a popular antioxidant and anti-inflammatory agent, resveratrol, improved glucose tolerance and reduced beta cell loss and oxidative stress in type 2 diabetes [45]. Furthermore, another group showed that quercetin, a flavonoid antioxidant, prevented and protected streptozotocin-induced oxidative stress and beta cell damage in rat pancreas [46]. Moreover, oxidative stress was also demonstrated to be the cause of apoptosis in IECs. Zhou et al. [47] have demonstrated that the p38 MAPK/PKC pathway plays an important role as a pro-apoptotic cellular signaling during intestinal epithelial cell damage induced by oxidative stress.

The analysis of miRNA expression revealed increased expression of miR-24 in IECs and PIs from EMS diagnosed horses. Lal et al. have shown that miR-24 inhibits cell proliferation by suppressing the expression of E2F2, MYC and other cell cycle regulatory genes [48]. This stands in good agreement with our data, as we observed that miR-24 over-expression correlates with decreased proliferation of IECs and PIs. Interestingly, IECs_EMS_ displayed enhanced expression of miR-223, which is involved in insulin signaling pathway [49]. Interestingly, miR-233 expression was enhanced in insulin resistant human adipose tissue [50] and in the insulin resistant heart [51]. Furthermore, expression of miR-223-3p was markedly increased in inflamed, compared with normal, IECs of both the small and large intestine in mice [52]. Thus it may be that EMS horses suffer from intestinal inflammation due to enhanced miR-233 expression. In PIs of EMS horses we have observed significantly up-regulated expression of miR-146; this correlates with the data of Lovis et al. [53], who revealed that insulin resistant β-cells are characterized by enhanced expression of miR-146 and increased apoptosis.

In the present study we have revealed that both IECs and PIs isolated from EMS individuals are characterized by deterioration in cellular physiology. Those cells displayed increased apoptosis, senescence, autophagy, mitochondrial function deterioration and finally ROS accumulation. The obtained findings suggest that antioxidants might exert therapeutic effects in the prevention and management of diabetes, and thus the supplementation of EMS horses’ diet with antioxidants seems to be highly reasonable and may alleviate EMS symptoms.

## 4. Materials and Methods

### 4.1. All Reagents Were Purchased in Sigma Aldrich (Saint Louis, Missouri, USA), Unless Otherwise Indicated 

#### 4.1.1. IEC Isolation and Culture

Tissue section was washed three times with Hank’s Balanced Salt Solution (HBSS) containing 2% gentamicin solution (10 ng/mL). The epithelium was separated from muscle tissue. Subsequently, intestines were minced into small fragments. The tissue biopsies were washed three times with Hank’s solution. The fragments were transferred into 2 mM EDTA prepared in a Hank’s solution (without Ca^2+^ and Mg^2+^) and incubated in a CO_2_ incubator at a temperature of 37 °C for 30 min. During incubation, tissues were strongly shaken. After incubation, the supernatants were collected and samples were centrifuged at 300× *g* for 4 min at room temperature. This process was repeated three times. For cell culture, pellets were suspended in Ham’s F-12 medium supplemented with 10% of fetal bovine serum and 1% of antibiotic/antimycotic solution (with 10,000 units penicillin, 10 mg streptomycin and 25 μg amphotericin B per mL, sterile-filtered). Cells were transferred into culture flasks (surface area 25 cm^2^) and maintained in a CO_2_ incubator (5% CO_2_ and 95% humidity) at a temperature of 37 °C. The medium was changed on average every two days. During the first three days only half of the medium was changed. When cultures reached 70% of confluence, the passage was performed using TrypLE™ solution (Life Technologies, Warsaw, Poland) according to the instructions of the supplier.

#### 4.1.2. Pancreatic Islets (PIs) Isolation and Culture

PIs were isolated from pancreas section harvested post mortem from healthy and EMS horses. First, tissue sections were washed extensively with HBSS with 1% P/S/A three times. Then, using surgical scissors and scalpel, specimens were minced and placed in collagenase type XI (4 mg/mL). Incubation with enzyme was performed at a temperature of 37 °C for 30 min, with gentle agitation of specimens every 10 min. Then, HBSS was poured into samples and they were centrifuged for 2 min at 290× *g*. Supernatant was discarded and, using needle tissue homogenate, was minced again and filtered with a 70 µm cell strainer. The remaining cells were re-suspended with HBSS and centrifuged again. Next, cell pellet in fresh HBSS was poured onto Histopaque 11100 and centrifuged for 18 min at 900× *g*. After centrifugation, cell suspension was filtered onto cell strainer again. PIs remaining on strainer were detached by washing with medium and seeded onto culture plates. Before attachment, cells were cultured in RPMI with 10% FBS and 1% P/S/A. When cells attached to the plate, the medium was changed to Dulbecco’s Modified Eagle Medium (DMEM). 

### 4.2. Phenotyping of IECs

The cells phenotype was investigated using flow cytometry. The analysis was performed on EqIECs from second passage. Specific monoclonal antibodies were used to detect the CD44 (R&D Systems, Minneapolis, MN, USA), CD45 (Novus Biologicals, Littleton, CO, USA), CD73b (R&D Systems, Minneapolis, MN, USA), CD90 and CD105 (both derived from Abcam, Cambridge, UK). Isotype-matched antibodies were used as controls. The concentration of antibodies used for analysis was 1:1000. Cells suspensions were incubated at a temperature of 4 °C for 30 min with the specific antibodies. The antibody for CD45 was conjugated with allophycocyanin (APC) and CD105 was conjugated with FITC. The unconjugated antibodies were detected using secondary antibody conjugated with Atto-488 (from Abcam, Cambridge, UK). The samples were incubated with secondary antibody for an additional 30 min at a temperature of 4 °C. At least 10,000 stained cells were acquired and analyzed by Becton Dickinson FACS Calibur (Franklin Lakes, NJ, USA) flow cytometer. The results of the measurements were analyzed using CellQuest Pro software (Franklin Lakes, NJ, USA). 

### 4.3. Detection of Mucin Complexes

The cultures of IECs were washed with HBSS, fixed with 10% formalin for 60 min and stained with 1% Alcian Blue solution (pH 2.5). Cells were incubated with the dye overnight at room temperature, avoiding exposure to excessive light. After staining cultures were washed in distilled water and observed using an AxioObserver.A1 inverted microscope (Zeiss, Oberkochen, Germany). Documentation of all cultures was performed using a PowerShot Camera (Canon, Tokyo, Japan).

### 4.4. Measurement of DNA Synthesis: BrdU Assay

DNA synthesis was assessed by measuring the incorporation of 5-bromo-2-deoxyuridine (BrdU) into cellular DNA. The test was performed after 72, 120 and 168 h of culture. The assay was performed using BrdU Cell Proliferation ELISA Kit (Abcam, Cambridge, UK) in accordance with the manufacturer’s instructions. After incubation with BrdU overnight at a temperature of 37 °C, cells were fixed and DNA was denaturated. Incorporation of BrdU was determined by incubation with anti-BrdU monoclonal antibody. Goat anti-mouse IgG conjugated with horseradish peroxidase (HRP) was used as a secondary antibody. Signal intensity was measured with a spectrophotometer plate reader (BMG Labtech, Ortenberg, Germany) at a wavelength of 450/550 nm.

### 4.5. Alamar Blue Viability Assay

Cell proliferation rate was established with resazurin-resorufin test. First, culture medium was replaced with a medium containing 10% of resazurin (Alamar Blue, Sigma Aldrich, Poznań, Poland). Next, cells were incubated with dye for 2 h at a temperature of 37 °C. Then, supernatants were collected and transferred to 96-well plates. Dye reduction was determined using a spectrometer (BMG Labtech, Ortenberg, Germany) at the specific wavelengths, i.e., 600 nm for resazurin and 690 nm as a background absorbance. A linear trendline equation was used to estimate cell numbers throughout the experiment.

### 4.6. Visualization of PIs Morphology and Senescence Markers

Cell morphology was assessed using an inverted epifluorescent microscope (Zeiss, Axio Observer A.1, Oberkochen, Germany). Briefly, using bright-field microscopy, cells were imaged after 72, 120 and 168 h of culture. To visualize senescence-associated β-galactosidase, we performed staining using Senescence Cells Histochemical Staining Kit following the manufacturer’s protocol. The amount of viable and dead cells were evaluated with Cellstain Double Staining Kit. Viable cells were stained with calcein AM and emitted green fluorescence, whereas dead cells’ nuclei were stained orange with propidium iodide. The percentage of dead cells was calculated.

### 4.7. Immunofluorescence Staining

Prior to staining, cells were seeded onto coverslips (Zeiss, Oberkochen, Germany). After 168 h of propagation, in order to visualize the mitochondria, cells were incubated with MitoRed dye (1:1000) at a temperature of 37 °C for 30 min. Then, cells were fixed with 4% paraformaldehyde (PFA) for 40 min at room temperature. Following three washing steps in HBSS, cells were then permeabilized with 0.2% Tween 20 in HBSS for 15 min at room temperature. After washing with HBSS three times, unspecific binding sites were blocked with blocking buffer (10% Goat Serum, 0.2% Tween-20 in HBSS) for 45 min. Cells were then incubated overnight at a temperature of 4 °C with primary antibodies against LAMP2 (Abcam, Cambridge, UK) or insulin diluted 1:500 in HBSS containing 1% Goat Serum and 0.2% Tween-20. Cells were then washed again and incubated for 1 h with goat anti-mouse secondary antibodies conjugated with atto-488 (dilution 1:1000, Abcam, Cambridge, UK), avoiding direct light. Subsequently, nuclei were counterstained by incubation with DAPI for 5 min. Then, actin filaments were stained using atto-488-labeled phalloidin at dilution 1:800 with HBSS for 40 min in the dark at room temperature and cells’ nuclei were counterstained with diamidino-2-phenylindole (DAPI; 1:1000) for 5 min. Coverslips were then mounted in ProLongGold Antifade (Life Technologies, Warsaw, Poland) and applied onto microscope slides. Specimens were observed and photographed using confocal microscope (Zeiss Cell Observer SD).

### 4.8. Superoxide Dismutase (SOD) Activity and Nitric Oxide (NO) Levels

Both SOD and NO levels were assessed after 168 h of propagation. To perform the tests, culture media was collected and frozen at a temperature of −20 °C prior to the experimental procedure. NO concentration was assessed using commercially available Griess reagent kit (Life Technologies, Warsaw, Poland). SOD activity was measured using a SOD Assay kit. All procedures were performed in accordance to the manufacturer’s instructions as described previously [54,55]. 

### 4.9. ROS Accumulation

In order to investigate ROS positive cells, the cells were incubated with an Oxidative Stress Kit (Merck, Darmstadt, Germany) and subjected to MUSE Cell Analyzer analysis. All procedures were performed following the manufacturer’ instructions.

### 4.10. Quantitative Real-Time Reverse Transcription Polymerase Chain Reaction (qRT-PCR)

After 168 h of culture, the cells were rinsed with HBSS and homogenized by TriReagent^®^, Sigma Aldrich, Poznań, Poland. Total RNA was isolated using phenol–chloroform method as previously described by Chomczynski & Sacchi [56]. The RNA was diluted in DEPC-treated water and analyzed using a nano-spectrometer (WPA Biowave II, Biochrom, Cambourne, Cambridge, UK). Genomic DNA digestion and cDNA synthesis were performed using PrimeScript kit (Takara, Clontech, Mountain View, CA, USA). For each reaction, 150 ng of total RNA was used. Both processes were performed in accordance with the manufacturers’ instructions using a T100 Thermal Cycler (Bio-Rad, Hercules, CA, USA). 

The qRT-PCR reactions were performed using a CFX ConnectTM Real-Time PCR Detection System (BioRad, Hercules, CA, USA). The reaction mixture contained 2 μL of cDNA in a total volume of 20 μL using SensiFast SYBR & Fluorescein Kit (Bioline, Cincinnati, OH, USA). Primer concentration in each reaction equaled 500 nM; primer sequences used in individual reactions are listed in Table 1. The average fold change in the gene expression of experimental cultures was compared with control cultures and calculated by the 2−DDCt method in relation to the housekeeping gene GAPDH.

### 4.11. The Analysis of miRNA Expression

The cDNA used for the detection of miRNA expression was synthesized from 500 ng of high-purity total RNA obtained by phenol-chloroform extraction method. For this purpose, the cells’ pellets were homogenized with 0.5 mL of trizol (TRI Reagent^®^). The RNA isolation procedure was performed according to the protocol supplied by the manufacturer. The Mir-X miRNA First-Strand Synthesis RT Kit (Clontech Laboratories, Inc., Mountain View, CA, USA) was used to obtain cDNA in the reverse transcriptase reaction. The quantity of miRNA transcript was investigated with real-time PCR technique. The total volume of PCR reaction was 20 μL. The reaction was performed using 0.5 μL of template, and the final concentration of primers was 0.4 μM. The following cycling conditions were applied during the reaction: a temperature of 95 °C for 10 s, followed by 35 cycles at a temperature of 95 °C for 5 s and annealing temperature of 57.4 °C for 20 s with a single fluorescence measurement. The list of miR specific primers used in the reaction is presented in the Table 2. The mRQ 3′ primer and U6snRNA primers were provided with the RT kit. The average fold change in the gene expression of experimental cultures was compared with control cultures and calculated by the 2−DDCt method in relation to U6snRNA.

## Figures and Tables

**Table 1:** Sequences of primers used in qPCR. Sequences, amplicon length and accession numbers of the primer sets. LC3: microtubule associated protein 1 light chain 3 beta (MAP1LC3B); Beclin: beclin 1, autophagy related (BECN1); LAMP2: lysosomal-associated membrane protein 2; GADPH: Glyceraldehyde-3-Phosphate Dehydrogenase; CHOP: DNA damage inducible transcript 3 (DDIT3); PERK: eukaryotic translation initiation factor 2-α kinase 3; PARKIN: parkin ligase; p53: tumor suppressor p53; p21: Cyclin-dependent kinase inhibitor 1A.

Gene	Primer	Sequence 5′-3′	Amplicon Length (bp)	Accesion No.
LC3	F:	TTACTGCTTTGCTCTGCCAC	213	XM_005608485.2
	R:	AGCTGCTTCTCCCCCTTGT		
Beclin	F:	GATGCGTTATGCCCAGATGC	147	XM_014729146.1
	R:	ATCCAGCGAACACTCTTGGG		
LAMP2	F:	GCACCCCTGGGAAGTTCTTA	139	XM_014733098.1
	R:	TTCGAGGATCTGTGCCAATCA		
GAPDH	F:	GATGCCCCAATGTTTGTGA	250	NM_001163856.1
	R:	AAGCAGGGATGATGTTCTGG		
CHOP	F:	AGCCAAAATCAGAGCCGGAA	272	XM_014844003.1
	R:	GGGGTCAAGAGTGGTGAAGG		
PERK	F:	GTGACTGCAATGGACCAGGA	283	XM_014852775.1
	R:	TCACGTGCTCACGAGGATATT		
PARKIN	F:	TCCCAGTGGAGGTCGATTCT	218	XM_014858374.1
	R:	CCCTCCAGGTGTGTTCGTTT		
p53	F:	TACTCCCCTGCCCTCAACAA	252	U37120.1
	R:	AGGAATCAGGGCCTTGAGGA		
p21	F:	GAAGAGAAACCCCCAGCTCC	241	XM_003365840.2
	R:	TGACTGCATCAAACCCCACA		

**Table 2:** The list of miRNA specific primers used in the reaction.

miRNA	Sequence of Specific Primer	Accesion Number of Sequence
miR-140-3p	TACCACAGGGTAGAACCACGGA	MIMAT0012926
h/m/r-miR-146a-5p	TGAGAACTGAATTCCATGGGTT	MI0012809
h/m/r-miR-223-3p	TGTCAGTTTGTCAAATACCCCA	MI0012953
h/e/m/r-miR-17-5p	CAAAGTGCTTACAGTGCAGGTAG	MI0012831
h/e/m/r-miR-24-3p	TGGCTCAGTTCAGCAGGAACAG	MI0012738
h/e/m/r-miR-7a-5p	TGGAAGACTAGTGATTTTGTTGT	MIMAT001290

## References

[1] Frank N. (2009). Equine Metabolic Syndrome. J. Equine Vet. Sci..

[2] Frank N. (2011). Equine metabolic syndrome. Vet. Clin. N. Am. Equine Pract..

[3] Marycz K., Michalak I., Kornicka K. (2018). Advanced nutritional and stem cells approaches to prevent equine metabolic syndrome. Res. Vet. Sci..

[4] Nawrocka D., Kornicka K., Śmieszek A., Marycz K. (2017). Spirulina platensis Improves Mitochondrial Function Impaired by Elevated Oxidative Stress in Adipose-Derived Mesenchymal Stromal Cells (ASCs) and Intestinal Epithelial Cells (IECs), and Enhances Insulin Sensitivity in Equine Metabolic Syndrome (EMS) Horses. Mar. Drugs.

[5] Basinska K., Marycz K., Śmieszek A., Nicpoń J. (2015). The production and distribution of IL-6 and TNF-α in subcutaneous adipose tissue and their correlation with serum concentrations in Welsh ponies with equine metabolic syndrome. J. Vet. Sci..

[6] Marycz K., Kornicka K., Basinska K., Czyrek A. (2016). Equine Metabolic Syndrome Affects Viability, Senescence, and Stress Factors of Equine Adipose-Derived Mesenchymal Stromal Stem Cells: New Insight into EqASCs Isolated from EMS Horses in the Context of Their Aging. Oxid. Med. Cell. Longev..

[7] Kornicka K., Houston J., Marycz K. (2018). Dysfunction of Mesenchymal Stem Cells Isolated from Metabolic Syndrome and Type 2 Diabetic Patients as Result of Oxidative Stress and Autophagy may Limit Their Potential Therapeutic Use. Stem Cell Rev. Rep..

[8] Kaniuk N.A., Kiraly M., Bates H., Vranic M., Volchuk A., Brumell J.H. (2007). Ubiquitinated-protein aggregates form in pancreatic beta-cells during diabetes-induced oxidative stress and are regulated by autophagy. Diabetes.

[9] Matveyenko A.V., Butler P.C. (2008). Relationship between beta-cell mass and diabetes onset. Diabetes Obes. Metab..

[10] Kasuga M. (2006). Insulin resistance and pancreatic β cell failure. J. Clin. Investig..

[11] Augstein P., Elefanty A.G., Allison J., Harrison L.C. (1998). Apoptosis and beta-cell destruction in pancreatic islets of NOD mice with spontaneous and cyclophosphamide-accelerated diabetes. Diabetologia.

[12] Silva J.P., Köhler M., Graff C., Oldfors A., Magnuson M.A., Berggren P.O., Larsson N.G. (2000). Impaired insulin secretion and beta-cell loss in tissue-specific knockout mice with mitochondrial diabetes. Nat. Genet..

[13] He C., Klionsky D.J. (2009). Regulation mechanisms and signaling pathways of autophagy. Annu. Rev. Genet..

[14] Chen Z., Li Y., Han J., Wang J., Yin J., Li J., Tian H. (2011). The double-edged effect of autophagy in pancreatic beta cells and diabetes. Autophagy.

[15] Kiyono K., Suzuki H.I., Matsuyama H., Morishita Y., Komuro A., Kano M.R., Sugimoto K., Miyazono K. (2009). Autophagy is activated by TGF-beta and potentiates TGF-beta-mediated growth inhibition in human hepatocellular carcinoma cells. Cancer Res..

[16] Fujitani Y., Kawamori R., Watada H. (2009). The role of autophagy in pancreatic beta-cell and diabetes. Autophagy.

[17] Marycz K., Kornicka K., Grzesiak J., Mieszek A., Apka J. Macroautophagy and Selective Mitophagy Ameliorate Chondrogenic Differentiation Potential in Adipose Stem Cells of Equine Metabolic Syndrome: New Findings in the Field of Progenitor Cells Differentiation. https://www.hindawi.com/journals/omcl/2016/3718468/.

[18] Marycz K., Kornicka K., Marędziak M., Golonka P., Nicpoń J. (2016). Equine metabolic syndrome impairs adipose stem cells osteogenic differentiation by predominance of autophagy over selective mitophagy. J. Cell. Mol. Med..

[19] Marycz K., Kornicka K., Irwin-Houston J.M., Weiss C. (2018). Combination of resveratrol and 5-azacytydine improves osteogenesis of metabolic syndrome mesenchymal stem cells. J. Cell. Mol. Med..

[20] Marycz K., Weiss C., Śmieszek A., Kornicka K. (2018). Evaluation of Oxidative Stress and Mitophagy during Adipogenic Differentiation of Adipose-Derived Stem Cells Isolated from Equine Metabolic Syndrome (EMS) Horses. Stem Cells Int..

[21] Marycz K., Kornicka K., Szlapka-Kosarzewska J., Weiss C. (2018). Excessive Endoplasmic Reticulum Stress Correlates with Impaired Mitochondrial Dynamics, Mitophagy and Apoptosis, in Liver and Adipose Tissue, but Not in Muscles in EMS Horses. Int. J. Mol. Sci..

[22] Ozcan U., Cao Q., Yilmaz E., Lee A.-H., Iwakoshi N.N., Ozdelen E., Tuncman G., Görgün C., Glimcher L.H., Hotamisligil G.S. (2004). Endoplasmic reticulum stress links obesity, insulin action, and type 2 diabetes. Science.

[23] Oyadomari S., Koizumi A., Takeda K., Gotoh T., Akira S., Araki E., Mori M. (2002). Targeted disruption of the Chop gene delays endoplasmic reticulum stress-mediated diabetes. J. Clin. Investig..

[24] Lenzen S., Drinkgern J., Tiedge M. (1996). Low antioxidant enzyme gene expression in pancreatic islets compared with various other mouse tissues. Free Radic. Biol. Med..

[25] Baynes J.W., Thorpe S.R. (1999). Role of oxidative stress in diabetic complications: A new perspective on an old paradigm. Diabetes.

[26] Robertson R.P. (2004). Chronic Oxidative Stress as a Central Mechanism for Glucose Toxicity in Pancreatic Islet Beta Cells in Diabetes. J. Biol. Chem..

[27] Peterson L.W., Artis D. (2014). Intestinal epithelial cells: Regulators of barrier function and immune homeostasis. Nat. Rev. Immunol..

[28] Wyse C.A., McNie K.A., Tannahill V.J., Tannahil V.J., Murray J.K., Love S. (2008). Prevalence of obesity in riding horses in Scotland. Vet. Rec..

[29] Leahy J.L., Bonner-Weir S., Weir G.C. (1992). Beta-cell dysfunction induced by chronic hyperglycemia. Current ideas on mechanism of impaired glucose-induced insulin secretion. Diabetes Care.

[30] Ferrannini E. (1998). Insulin resistance versus insulin deficiency in non-insulin-dependent diabetes mellitus: Problems and prospects. Endocr. Rev..

[31] Eizirik D.L. (1996). Beta-cell defence and repair mechanisms in human pancreatic islets. Horm. Metab. Res..

[32] Donath M.Y., Gross D.J., Cerasi E., Kaiser N. (1999). Hyperglycemia-induced beta-cell apoptosis in pancreatic islets of Psammomys obesus during development of diabetes. Diabetes.

[33] Elmore S. (2007). Apoptosis: A Review of Programmed Cell Death. Toxicol. Pathol..

[34] Moley K.H., Chi M.M., Knudson C.M., Korsmeyer S.J., Mueckler M.M. (1998). Hyperglycemia induces apoptosis in pre-implantation embryos through cell death effector pathways. Nat. Med..

[35] Liu Z., Lv Y., Zhao N., Guan G., Wang J. (2015). Protein kinase R-like ER kinase and its role in endoplasmic reticulum stress-decided cell fate. Cell Death Dis..

[36] Karaskov E., Scott C., Zhang L., Teodoro T., Ravazzola M., Volchuk A. (2006). Chronic palmitate but not oleate exposure induces endoplasmic reticulum stress, which may contribute to INS-1 pancreatic beta-cell apoptosis. Endocrinology.

[37] Behrends C., Sowa M.E., Gygi S.P., Harper J.W. (2010). Network organization of the human autophagy system. Nature.

[38] Back S.H., Scheuner D., Han J., Song B., Ribick M., Wang J., Gildersleeve R.D., Pennathur S., Kaufman R.J. (2009). Translation attenuation through eIF2alpha phosphorylation prevents oxidative stress and maintains the differentiated state in beta cells. Cell Metab..

[39] Kaneto H., Katakami N., Matsuhisa M., Matsuoka T., Kaneto H., Katakami N., Matsuhisa M., Matsuoka T. (2010). Role of Reactive Oxygen Species in the Progression of Type 2 Diabetes and Atherosclerosis, Role of Reactive Oxygen Species in the Progression of Type 2 Diabetes and Atherosclerosis. Mediat. Inflamm. Mediat. Inflamm..

[40] Frei B. (1994). Reactive oxygen species and antioxidant vitamins: Mechanisms of action. Am. J. Med..

[41] Busik J.V., Mohr S., Grant M.B. (2008). Hyperglycemia-Induced Reactive Oxygen Species Toxicity to Endothelial Cells Is Dependent on Paracrine Mediators. Diabetes.

[42] Sakuraba H., Mizukami H., Yagihashi N., Wada R., Hanyu C., Yagihashi S. (2002). Reduced beta-cell mass and expression of oxidative stress-related DNA damage in the islet of Japanese Type II diabetic patients. Diabetologia.

[43] Kaneto H., Kajimoto Y., Fujitani Y., Matsuoka T., Sakamoto K., Matsuhisa M., Yamasaki Y., Hori M. (1999). Oxidative stress induces p21 expression in pancreatic islet cells: Possible implication in beta-cell dysfunction. Diabetologia.

[44] Piro S., Anello M., Di Pietro C., Lizzio M.N., Patanè G., Rabuazzo A.M., Vigneri R., Purrello M., Purrello F. (2002). Chronic exposure to free fatty acids or high glucose induces apoptosis in rat pancreatic islets: Possible role of oxidative stress. Metab. Clin. Exp..

[45] Lee Y.-E., Kim J.-W., Lee E.-M., Ahn Y.-B., Song K.-H., Yoon K.-H., Kim H.-W., Park C.-W., Li G., Liu Z. (2012). Chronic resveratrol treatment protects pancreatic islets against oxidative stress in db/db mice. PLoS ONE.

[46] Coskun O., Kanter M., Korkmaz A., Oter S. (2005). Quercetin, a flavonoid antioxidant, prevents and protects streptozotocin-induced oxidative stress and β-cell damage in rat pancreas. Pharmacol. Res..

[47] Zhou Y., Wang Q., Evers B.M., Chung D.H. (2006). Oxidative Stress-Induced Intestinal Epithelial Cell Apoptosis is Mediated By p38 Mapk. Biochem. Biophys. Res. Commun..

[48] Lal A., Navarro F., Maher C., Maliszewski L.E., Yan N., O’Day E., Chowdhury D., Dykxhoorn D.M., Tsai P., Hofman O. (2009). miR-24 inhibits cell proliferation by suppressing expression of E2F2, MYC and other cell cycle regulatory genes by binding to “seedless” 3′UTR microRNA recognition elements. Mol. Cell.

[49] Chakraborty C., Doss C.G.P., Bandyopadhyay S., Agoramoorthy G. (2014). Influence of miRNA in insulin signaling pathway and insulin resistance: Micro-molecules with a major role in type-2 diabetes. Wiley Interdiscip. Rev. RNA.

[50] Chuang T.-Y., Wu H.-L., Chen C.-C., Gamboa G.M., Layman L.C., Diamond M.P., Azziz R., Chen Y.-H. (2015). MicroRNA-223 Expression Is Upregulated in Insulin Resistant Human Adipose Tissue. J. Diabetes Res..

[51] Lu H., Buchan R.J., Cook S.A. (2010). MicroRNA-223 regulates Glut4 expression and cardiomyocyte glucose metabolism. Cardiovasc. Res..

[52] Lee J., Park E.J., Yuki Y., Ahmad S., Mizuguchi K., Ishii K.J., Shimaoka M., Kiyono H. (2015). Profiles of microRNA networks in intestinal epithelial cells in a mouse model of colitis. Sci. Rep..

[53] Lovis P., Roggli E., Laybutt D.R., Gattesco S., Yang J.-Y., Widmann C., Abderrahmani A., Regazzi R. (2008). Alterations in microRNA expression contribute to fatty acid-induced pancreatic beta-cell dysfunction. Diabetes.

[54] Kornicka K., Marycz K., Tomaszewski K.A., Marędziak M., Śmieszek A. (2015). The Effect of Age on Osteogenic and Adipogenic Differentiation Potential of Human Adipose Derived Stromal Stem Cells (hASCs) and the Impact of Stress Factors in the Course of the Differentiation Process. Oxid. Med. Cell. Longev..

[55] Kornicka K., Marycz K., Marędziak M., Tomaszewski K.A., Nicpoń J. (2017). The effects of the DNA methyltranfserases inhibitor 5-Azacitidine on ageing, oxidative stress and DNA methylation of adipose derived stem cells. J. Cell. Mol. Med..

[56] Chomczynski P., Sacchi N. (1987). Single-step method of RNA isolation by acid guanidinium thiocyanate-phenol-chloroform extraction. Anal. Biochem..

